# Modulation of circulating inflammatory biomarkers by SGLT2 inhibitors in diabetic patients with acute myocardial infarction: A quantitative meta-analysis of laboratory evidence

**DOI:** 10.5937/jomb0-65034

**Published:** 2026-06-16

**Authors:** Jia Xu, Yuanhong Jie, Tao Dong, Qi Li, Xiaobing Liu

**Affiliations:** 1 Beijing Hospital of Integrated Traditional Chinese and Western Medicine, Department of Pharmacy, Beijing, China; 2 Hospital of Jiangxi Provincial Armed Police Corps, Nanchang, China; 3 Beijing Hospital of Integrated Traditional Chinese and Western Medicine, Department of Pathology, Beijing, China; 4 Huidong People's Hospital, Department of Endocrinology, Huizhou, China

**Keywords:** SGLT2 inhibitors, inflammatory biomarkers, IL-6, hs-CRP, acute myocardial infarction, diabetes mellitus, meta-analysis, SGLT2 inhibitori, inflamatorni biomarkeri, IL-6, hs-CRP, akutni infarkt miokarda, dijabetes melitus, meta-analiza

## Abstract

**Background:**

Acute myocardial infarction (AMI) in patients with type 2 diabetes mellitus (T2DM) is characterized by an exaggerated inflammatory response and adverse myocardial remodeling. Sodium-glucose cotransporter-2 inhibitors (SGLT2-I) have demonstrated cardiometabolic benefits; however, their effects on circulating inflammatory biomarkers in diabetic AMI remain inconsistent. This meta-analysis aimed to quantitatively evaluate laboratory-based evidence regarding the modulation of key inflammatory markers by SGLT2-I therapy in this high-risk population.

**Methods:**

A systematic search of PubMed, ScienceDirect, Cochrane Library, Web of Science, CNKI, and Wanfang databases was conducted up to December 2025. Randomized controlled trials and prospective cohort studies involving T2DM patients with AMI were included. Primary laboratory endpoints were circulating inflammatory biomarkers (interleukin-6 [IL-6], high-sensitivity C-reactive protein [hs-CRP], tumor necrosis factor-a [TNF-a]). Secondary outcomes included left ventricular ejection fraction (LVEF) and major adverse cardiovascular events (MACE).

## Abstract (sr)

**Uvod:**

Akutni infarkt miokarda (AIM) kod pacijenata sa dijabetesom melitusom tipa 2 (DM2) karakteriše se preuveličanim inflamatornim odgovorom i nepovoljnim remodeliranjem miokarda. Inhibitori natrijum-glukoznog kotransportera-2 (SGLT2-I) pokazali su kardiometaboličke koristi; međutim, njihovi efekti na cirkulišuće inflamatorne biomarkere kod dijabetičkog AIM ostaju nedosledni. Ova meta-analiza imala je za cilj kvantitativnu procenu laboratorijskih dokaza u vezi sa modulacijom ključnih inflamatornih markera terapijom SGLT2-I kod ove populacije visokog rizika.

**Metode:**

Sistematska pretraga baza podataka PubMed, ScienceDirect, Cochrane Library, Web of Science, CNKI i Wanfang sprovedena je do decembra 2025. godine. Uključena su randomizovana kontrolisana ispitivanja i prospektivne kohortne studije koje su uključivale pacijente sa dijabetesom tipa 2 sa akutnim infarktom miokarda. Primarni laboratorijski krajnji ciljevi bili su cirkulišući inflamatorni biomarkeri (interleukin-6 [IL-6], C-reaktivni protein visoke osetljivosti [hs-CRP], faktor nekroze tumora-a [TNF-a]). Sekundarni ishodi uključivali su ejekcionu frakciju leve komore (LVEF) i glavne neželjene kardiovaskularne događaje (MACE).

**Rezultati:**

Uključeno je trinaest studija koje su obuhvatile 2.453 pacijenta. Meta-analiza je pokazala da je, u poređenju sa kontrolnom grupom koja nije primala SGLT2 inhibitore, posmatračka grupa lečena SGLT2 inhibitorima pokazala značajno povišen nivo LVEF (MD=2,46, 95%CI: 1,04 do 3,87; P&lt;0,001), kao i značajno smanjene nivoe IL-6 (MD=-2,33, 95%CI: -4,29 do -0,37; P&lt;0,001) i hs-CRP)(MD=-1,90, 95%CI: -2,30 do -1,49; P&lt;0,001), i značajno manji rizik od MACE (RR=0,47, 95%CI: 0,36 do 0,61; P&lt;0,001). Pored toga, nije primećeno značajno smanjenje nivoa TNF-a u grupi koja je primala SGLT2-I u poređenju sa grupom koja nije primala SGLT2-I (MD = -2,66, 95% CI: -6,66 do 1,33; P = 0,19), potencijalno zbog heterogenosti između studija i ograničene veličine uzorka. Ukupan kvalitet dokaza je ocenjen kao umeren.

**Zaključak:**

Ova meta-analiza pruža laboratorijske dokaze da je terapija inhibitorima SGLT2 povezana sa značajnim smanjenjem nivoa cirkulišućeg IL-6 i hs-CRP kod dijabetičara sa akutnim infarktom miokarda, što podržava potencijalni antiinflamatorni mehanizam koji prevazilazi snižavanje glukoze. Ovi nalazi ističu translacioni značaj inflamatornih biomarkera u proceni kardiometaboličkih terapijskih strategija i naglašavaju potrebu za većim mehanističkim studijama kako bi se dalje razjasnili uključeni biohemijski putevi.

## Introduction

Acute myocardial infarction (AMI) is one of the leading causes of death worldwide, and diabetes mellitus (DM) constitutes a well-established risk factor for AMI. Consequently, the comorbidity of DM and AMI is common in clinical practice [1]. Studies have indicated that the presence of DM increases the risk of heart failure following myocardial infarction by 30% to 42%. Moreover, within the first month after AMI onset, the mortality rate is 20.2% in non-diabetic patients, whereas it reaches as high as 42.0% in those with concurrent DM [2][3]. Therefore, patients with DM complicated by AMI exhibit a poor prognosis, posing significant challenges to clinical management. After myocardial infarction, tissue injury rapidly activates the immune system and triggers an inflammatory response. Proinflammatory cytokines released by the infarcted myocardium can activate leukocyte integrins, thereby recruiting a large number of inflammatory cells to infiltrate the infarcted area. While moderate inflammation promotes the clearance of necrotic myocardial tissue and ventricular remodeling, excessive inflammatory responses exacerbate tissue damage and impede cardiac repair processes [4]. Notably, patients with type 2 diabetes mellitus demonstrate enhanced inflammasome activation due to hyperglycemia, insulin resistance, and oxidative stress, rendering them susceptible to exaggerated inflammatory responses during AMI [5]. Thus, regulating the inflammatory response has emerged as a crucial potential therapeutic target for improving the prognosis of patients with DM and AMI. Sodium-glucose cotransporter-2 inhibitors (SGLT2-I) are regarded as the preferred agents for T2DM patients with established atherosclerotic cardiovascular disease [6]. Beyond their significant hypoglycemic effects, SGLT2-I have also been shown to effectively inhibit inflammation [7]. However, conflicting evidence exists: a study reported that luseogliflozin treatment for 12 weeks failed to improve the levels of inflammatory markers [8]. Currently, clinical research findings regarding the impact of SGLT2-I on inflammatory factors remain inconsistent, and the reliability of conclusions awaits further verification. Therefore, this study aims to conduct a meta-analysis to evaluate the effects of SGLT2-I on inflammatory factor levels in patients with DM complicated by AMI.

## Materials and methods

### Literature retrieval strategy

China National Knowledge Infrastructure (CNKI), Wanfang, PubMed, Science Direct, The Cochrane Library, Web of science and other databases were searched from January 1997 to December 2025. Retrieval is performed in a combination of subject words and free words. Keywords: SGLT-2 inhibitors, diabetes, acute myocardial infarction, empagliflozin, canagliflozin, dapagliflozin, gagliflozin, luseogliflozin, sodium-glucose co-transporter-2 inhibitors, ST elevation myocardial infarction, non-ST-elevation myocardial infarction, IL-6, hs-CRP TNF-a, inflammation, MACE, LVEF, etc. The search strategies for each database were as follows:

PubMed: (SGLT2 inhibitor [Title/Abstract]OR Sodium-Glucose Transporter 2 Inhibitors [Title/Abstract] OR Sodium-Glucose Co-Transporter 2 Inhibitors [Title/Abstract] OR empagliflozin [Title/Abstract] Or canagliflozin [Title/Abstract] OR dapagliflozin [Title/Abstract] OR luseogliflozin [Title/Abstract] OR SGLT-2 [Title/Abstract] OR SGLT2 [Title/Abstract]) AND (Diabetes Mellitus[Title/Abstract] OR Diabetes [Title/Abstract] OR Type 2 Diabetes Mellitus [Title/Abstract] OR T2DM [Title/Abstract]) AND (Acute Myocardial Infarction [Title/Abstract] OR AMI [Title/Abstract] OR Myocardial Infarction [Title/Abstract] OR ST Elevation Myocardial Infarction [Title/Abstract] OR STEMI [Title/Abstract] OR Non-ST-Elevation Myocardial Infarction [Title/Abstract] OR NSTEMI [Title/Abstract]) AND (Interleukin-6 [Title/Abstract] OR IL-6 [Title/Abstract] OR C-Reactive Protein [Title/Abstract] OR CRP [Title/Abstract] OR High-Sensitivity C-Reactive Protein [Title/Abstract] OR hs-CRP [Title/Abstract] OR Tumor Necrosis Factor-alpha [Title/Abstract] OR TNF-alpha [Title/Abstract] OR Tumor Necrosis Factor-a [Title/Abstract] OR TNF-a [Title/Abstract] OR Inflammation [Title/Abstract]) AND (Major Adverse Cardiac Events [Title/Abstract] OR MACE [Title/Abstract] OR Left Ventricular Ejection Fraction [Title/Abstract] OR LVEF [Title/Abstract]).

Web of Science: TS = (SGLT2 inhibitor OR SGLT-2 inhibitor OR »sodium-glucose co-transporter 2 inhibitor« OR empagliflozin OR canagliflozin OR dapagliflozin OR ipragliflozin OR luseogliflozin) AND TS = (diabetes OR »diabetes mellitus« OR T2DM) AND TS = (acute myocardial infarction OR AMI OR STEMI OR »ST-segment elevations myocardial infarction« OR NSTEMI OR »non-ST-segment elevation myocardial infarction«) AND TS = (IL-6 OR »Interleukin-6« OR hs-CRP OR »high-sensitivity C-reactive protein« OR TNF-a OR »tumor necrosis factor-alpha« OR inflammation OR MACE OR »major adverse cardiac events« OR LVEF OR »left ventricular ejection fraction«).

Science Direct: SGLT2 inhibitor*:ti OR SGLT-2 inhibitor*:ti OR sodium-glucose co-transporter 2 inhibitor*:ti OR empagliflozin*:ti OR canagliflozin*:ti OR dapagliflozin*:ti OR luseogliflozin*:ti AND diabetes*:ti OR diabetes mellitus*:ti OR T2DM*:ti AND acute myocardial infarction*:ti OR STEMI*:ti OR NSTEMI*:ti OR non-ST-segment elevation myocardial infarction*:ti AND IL-6*:ti OR hs-CRP*:ti Or TNF-a*:ti OR tumor necrosis factor-alpha*:ti OR inflammation OR major adverse cardiac events*:ti OR left ventricular ejection fraction*:ti.

The Cochrane Library: #1 (SGLT2 inhibitor OR SGLT-2 inhibitor OR sodium-glucose co-transporter 2 inhibitor OR empagliflozin OR canagliflozin OR dapagliflozin OR ipragliflozin OR luseogliflozin).#2 (diabetes OR diabetes mellitus OR T2DM).#3 (acute myocardial infarction OR AMI OR STEMI OR ST-segment elevation myocardial infarction OR NSTEMI OR non-ST-segment elevation myocardial infarction).#4 (IL-6 OR Interleukin-6 OR hs-CRP OR high-sensitivity C-reactive protein OR TNF-a OR tumor necrosis factor-alpha OR inflammation OR MACE OR major adverse cardiac events OR LVEF OR left ventricular ejection fraction).#5 #1 AND #2 AND #3 AND #4.

CNKI: (Topic: SGLT2 inhibitors OR Topic: SGLT-2 inhibitors OR Topic: Sodium-glucose co-transporter 2 inhibitors OR Topic: Empagliflozin OR Topic: Canagliflozin OR Topic: Dapagliflozin OR Topic: Luseogliflozin) AND (Topic: Diabetes mellitus OR Topic: Type 2 diabetes mellitus) AND (Topic: Acute myocardial infarction OR Topic: AMI OR Topic: STEMI OR Topic: ST-segment elevation myocardial infarction OR Topic: NSTEMI OR Topic: Non-ST-segment elevation myocardial infarction) AND (Topic:Interleukin-6 OR Topic: High-sensitivity C-reactive protein OR Topic: Tumor necrosis factor-a OR Topic: Inflammation OR Topic: Major adverse cardiovascular events OR Topic: Left ventricular ejection fraction).

Wanfang: Title/Keyword:SGLT2 inhibitors OR Title/Keyword:SGLT-2 inhibitors OR Title/Keyword: Sodium-glucose co-transporter 2 inhibitors OR Title/Keyword: Empagliflozin OR Title/Keyword:Canagliflozin OR Title/Keyword: Dapagliflozin OR Title/Keyword: Luseogliflozin AND Title/Keyword: Diabetes mellitus OR Title/Keyword: Type 2 diabetes mellitus AND Title/Keyword: Acute myocardial infarction OR Title/Keyword: AMI OR Title/Keyword: ST-segment elevation myocardial infarction OR Title/Keyword: Non-ST-segment elevation myocardial infarction AND Title/Keyword: Interleukin-6 OR Title/Keyword: High-sensitivity C-reactive protein OR Title/Keyword: Tumor necrosis factor-a OR Title/Keyword: Inflammatory factors OR Title/Keyword: Major adverse cardiovascular events OR Title/Keyword: Left ventricular ejection fraction.

### Inclusion and exclusion criteria

Inclusion criteria: (1) The subjects were patients with type 2 DM complicated with AMI; (2) The type of study was published randomized controlled trial (RCT) or prospective cohort study; (3) The intervention measures were as follows: the observation group received SGLT2-I treatment, while the control group did not receive SGLT2-I treatment or placebo treatment; (4) Outcome indicators: At least one of the following indicators after treatment is included: 1 left ventricular ejection fraction; 2 MACE; 3 Inflammatory factors: IL-6, hs-CRP TNF-a.

Exclusion criteria: (1) repeated published literature; (2) Conference abstract, review, system analysis, animal experiments, case reports, master thesis; (3) The subjects were patients with type 1 DM, gestational diabetes or other special types of diabetes or other cardiovascular diseases; (4) The full text cannot be obtained; (5) Poor quality, unclear data or unable to obtain full-text literature; (6) Non-Chinese and English literature; (7) The course of treatment is not clear.

### Literature screening and data extraction

Two evaluators independently screened the literature, extracted the data and cross-checked. In case of disagreement, a third party was consulted to assist in the judgment. The lack of data was supplemented by contacting the author as much as possible. The following data were extracted: name of the first author, year of publication, sample size, age, intervention method, intervention time and outcome index.

### Bias risk assessment

Two evaluators independently evaluated the risk of bias of the included studies according to the bias risk assessment tool of the Cochrane evaluation manual for RCTs. The methodological quality of prospective cohort studies was assessed by the Newcastle-Ottowa Scale (NOS), including three factors: patient selection, comparability of study groups, and outcome evaluation. The score range of each study was 0-9, ≥ 7 points were considered to be high quality, 5-6 points were medium quality studies, and ≤ 4 points were low quality studies.

### Statistical methods

Meta-analysis was performed using RevMan 5.3 software and stata MP The measurement data were expressed as mean difference (MD) or standardized mean difference (SMD) and its 95% confidence interval (CI). The heterogeneity was evaluated by c^2^ test combined with statistics I^2^. The Risk Ratio (RR) and 95% CI were used as the effect size indexes for the enumeration data. The heterogeneity test was evaluated by Q test and I^2^ value. If I^2^ ≤ 50% and P ≥ 0.10, it indicated that there was no statistical heterogeneity among the studies, and the fixed effect model was used. On the contrary, the random effect model was used and the sensitivity analysis was performed using the stepwise elimination method to evaluate the impact of each study on the overall results to verify the robustness of the meta-analysis results. GRADE was used to evaluate the quality of evidence. Test level a = 0.05.

### Subgroup analyses

Follow-up duration: Subgroup analyses were performed according to follow-up duration (<6 months vs. ≥6 months).

MACE components: Subgroup analyses were conducted by specific types of MACE, including angina pectoris, cardiogenic death, and heart failure.

Note: For any outcome indicator where the number of studies reporting stratified data was fewer than 3, the corresponding subgroup analysis was not performed due to insufficient statistical power, and only the pooled overall effect estimate was reported.

## Results

### Literature search and screening

**Figure 1:**
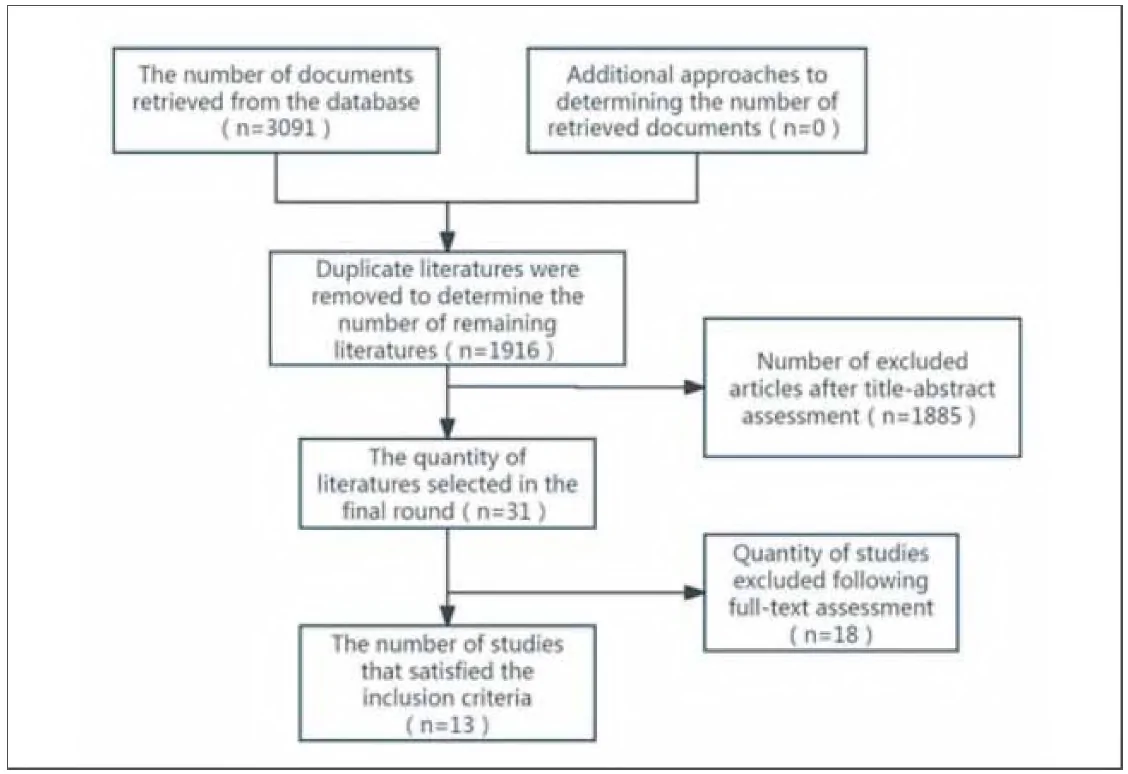
Literature retrieval flow chart.

A total of 3091 articles were obtained in the initial search (1218 in PubMed, 265 in Science Direct, 331 in Cochrane Library, 775 in web of science, 274 in CNKI, 228 in Wanfang). After screening by inclusion and exclusion criteria, 13 articles finally met the inclusion criteria of this meta-analysis. The process of research screening is shown in Figure 1.

### Basic characteristics of the included studies

A total of 13 studies were ultimately included in this meta-analysis, encompassing 2453 patients with DM complicated by AMI. Among these included studies, 2 were prospective cohort studies and 11 were RCTs. The basic characteristics of the included studies are summarized in Table 1 [9][10][11][12][13][14][15][16][17][18][19][20][21].

**Table 1:** Characteristics of patients included in the study.

Reference	Study<br>design	Treatment<br>Duration	non-SGLT2-I group			SGLT2-I group			Outcomes
			N	Age	Intervention	N	Age	Intervention	
WANG P<br>ZH 2025<br>[9]	RCT	6 months	60	65.24±<br>12.59	Sacubitril/<br>valsartan sodium<br>on the basis of<br>conventional<br>treatment	60	64.86±<br>11.72	Dapagliflozin<br>added to the<br>control group's<br>regimen	LVEF, MACE (Angina<br>pectoris, cardiogenic<br>death, non-fatal<br>myocardial infarction,<br>heart failure)
LIU CH<br>CH 2025<br>[10]	Prospective<br>Cohort<br>Study	6 months	88	64.6±<br>11.8	Non-SGLT-2<br>inhibitor hypoglycemic<br>agents<br>on the basis of<br>guideline-recommended<br>STEMI<br>treatment	92	61.6±<br>11.9	Dapagliflozin<br>treatment	MACE (Cardiogenic<br>death, unplanned<br>revascularization,<br>hospitalization for heart<br>failure, stroke)
TANG L<br>L2025<br>[11]	RCT	6 months	62	57.56±<br>8.12	Insulin plus original<br>oral<br>hypoglycemic agents	52	57.28±<br>8.42	Dapagliflozin<br>added to the<br>control group's<br>regimen	LVEF, MACE (Recurrent<br>myocardial infarction,<br>cardiovascular death,<br>stroke)
LIU K<br>2024<br>[12]	RCT	3 months	53	64.40±<br>6.94	Targeted treatment<br>per guidelines	55	62.72±<br>7.61	Dapagliflozin<br>added to the<br>control group's<br>regimen	LVEF, IL-6, TNF-a
LU Y L<br>2025<br>[13]	RCT	24 weeks	59	67.61±<br>5.21	Conventional<br>hypoglycemic<br>therapy	59	68.29±<br>5.09	Dapagliflozin<br>added to the<br>control group's<br>regimen	LVEF, MACE (Recurrent<br>myocardial infarction,<br>angina pectoris,<br>readmission for heart<br>failure, malignant<br>arrhythmia)
ZHAO<br>L 2022<br>[14]	RCT	6 months	49	59.38±<br>8.05	Insulin therapy<br>was performed on<br>the basis of routine<br>treatment.	49	59.67±<br>8.64	Dapagliflozin<br>added to the<br>control group's<br>regimen	hs-CRP IL-6, MACE<br>(Recurrent acute<br>myocardial infarction,<br>death, angina pectoris,<br>heart failure)
LIN W<br>2024<br>[15]	RCT	3 months	55	53.77±<br>5.14	conventional<br>therapy	59	54.58±<br>6.26	Dapagliflozin<br>added to the<br>control group's<br>regimen	LVEF, MACE (Cardiogenic<br>death, angina pectoris,<br>heart failure, recurrent<br>myocardial infarction)
QI Y<br>2024<br>[16]	RCT	6 months	47	60.42±<br>5.84	Conventional<br>standardized drug<br>therapy	53	61.27±<br>5.92	Empagliflozin<br>added to the<br>control group's<br>regimen	TNF-a, IL-6
LUO SH<br>J 2025<br>[17]	Prospective<br>Cohort<br>Study	1.8<br>(1.5—2.3)<br>years	718	64.9±<br>11.7	Basic<br>treatment	443	64.4±<br>11.5	SGLT2i added to<br>basic treatment	LVEF, MACE (Readmission<br>for heart failure, recurrent<br>myocardial infarction,<br>cardiogenic death)
Heshmat<br>A 2025<br>[18]	RCT	4 weeks	27	56±<br>6.4	Standard<br>treatment	27	52.1±<br>8.5	Standard treatment<br>combined<br>with dapagliflozin	LVEF
Lv Y<br>2024<br>[19]	RCT	6 months	47	63.92±<br>9.85	Conventional<br>treatment<br>combined with<br>sacubitril/valsartan<br>sodium	51	65.01±<br>10.21	Conventional<br>treatment +<br>sacubitril/valsartan<br>sodium +<br>dapagliflozin	LVEF, hs-CRP MACE<br>(Recurrent myocardial<br>infarction, heart failure,<br>angina pectoris,<br>cardiogenic death)
ZHANG<br>L F 2024<br>[20]	RCT	6 months	40	55.83±<br>8.83	Sacubitril/valsartan<br>treatment	40	56.23±<br>10.64	Sacubitril/valsartan<br>combined<br>with dapagliflozin	LVEF, MACE (Type not<br>clearly specified)
ZHANG T<br>CH 2022<br>[21]	RCT	3 months	56	66.00±<br>7.29	Conventional<br>standardized<br>treatment	52	66.02±<br>7.57	Canagliflozin<br>+ conventional<br>standardized<br>treatment	LVEF

### Quality evaluation

**Figure 2:**
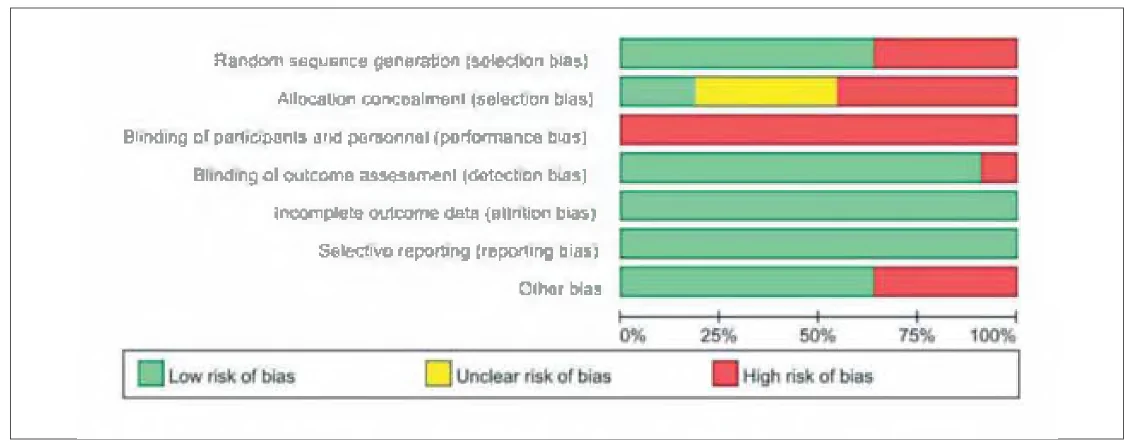
Risk of bias graph.

**Figure 3:**
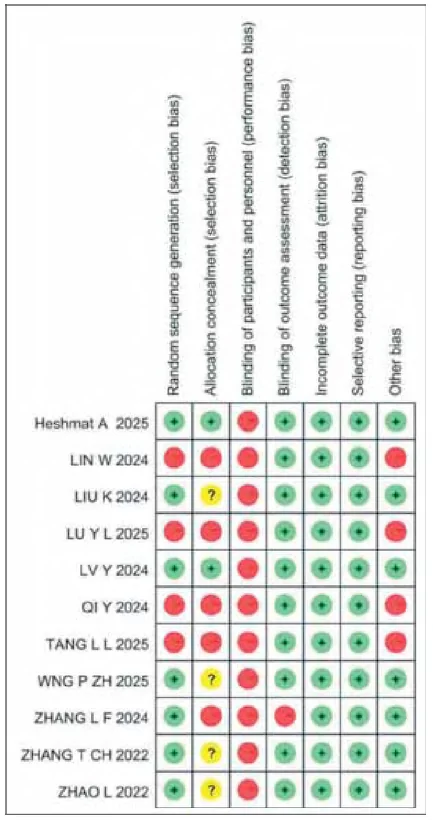
Risk of bias summary.

A total of 2 cohort studies [10][17] were included in this study, which were evaluated by NOS, and all of them were high-quality studies. A total of 11 randomized controlled trials [9][11][12][13][14][15][16][18][19][20][21] were included. The results of the Cochrane bias risk tool were presented in the risk of bias diagram (Figure 2) and the risk of bias summary diagram (Figure 3).

### Meta-analysis

### LVEF

**Figure 4:**
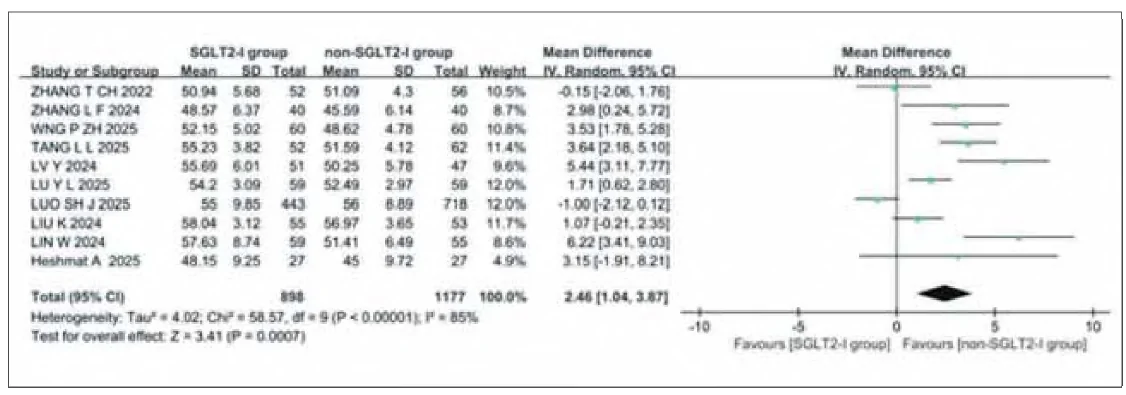
Forest plots regarding LVEF.

**Figure 5:**
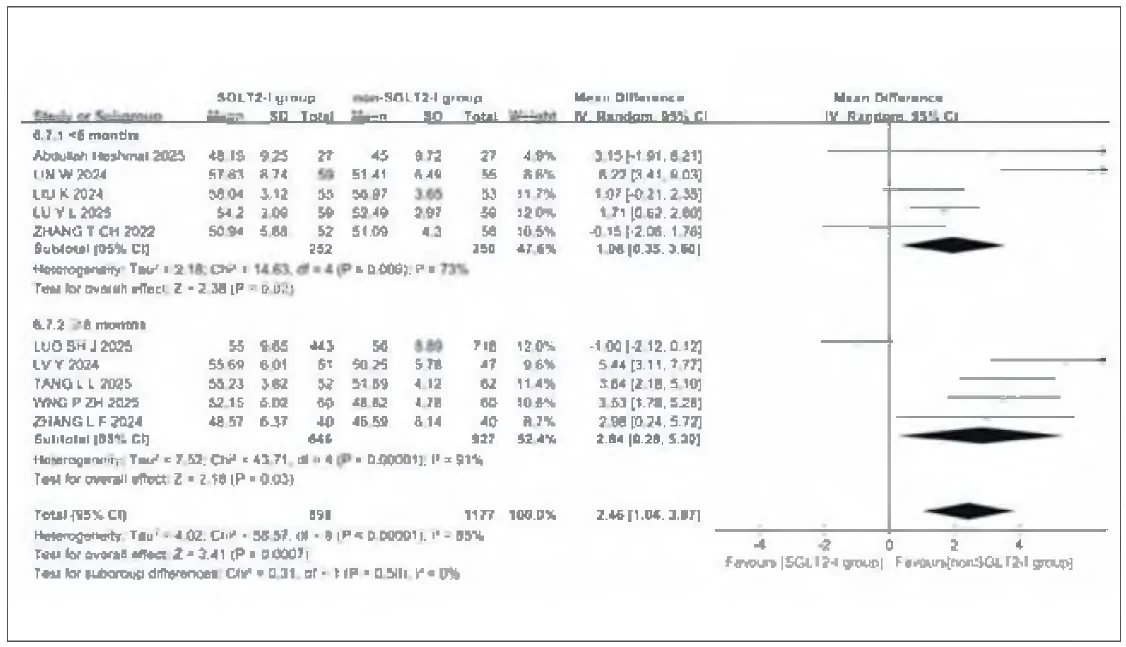
Forest plot of subgroup analysis for LVEF by follow-up duration.

10 studies reported LVEF, including 2075 patients (898 in SGLT2-I group and 1177 in non-SGLT2-I group). There was significant heterogeneity among studies (I^2^ = 85%, P < 0.001), so a random effect model was used for meta-analysis. The results showed that the use of SGLT2-I significantly increased LVEF levels (MD = 2.46, 95% CI = 1.04-3.87) compared with the non-use of SGLT2-I. P < 0.001; Figure 4). A further subgroup analysis was conducted according to follow-up duration (<6 months vs. >≥ months). The results demonstrated that compared with non-use of SGLT2-I, use of SGLT2-I was associated with a significant increase in LVEF both in the <6 months subgroup (MD = 1.98, 95%CI: 0.35-3.60; P=0.02) and in the ≥6 months subgroup (Md = 2.84, 95%CI: 0.28-5.39; P=0.03; Figure 5). Moreover, the magnitude of LVEF improvement was more pronounced in the >6 months subgroup than in the <6 months subgroup.

### IL-6

**Figure 6:**
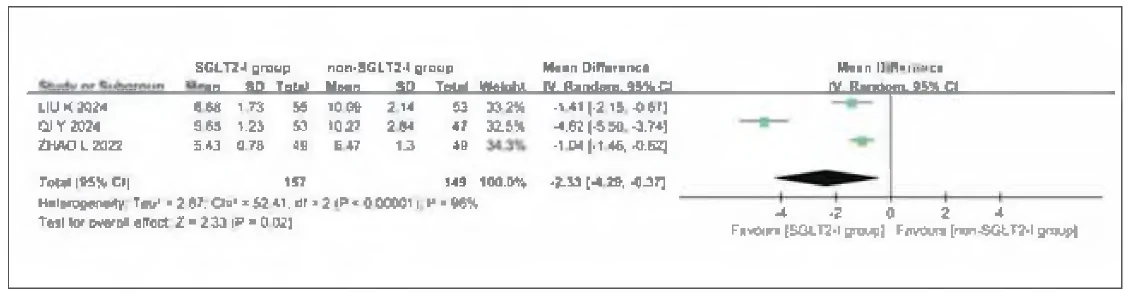
Forest plots regarding IL-6.

3 studies reported IL-6, including 306 patients (157 in SGLT2-I group and 149 in non-SGLT2-I group). There was significant heterogeneity among studies (I^2^ = 96%, P < 0.001), so a random effect model was used for meta-analysis. The results showed that the use of SGLT2-I could significantly reduce the level of IL-6 (MD = -2.33, 95% CI = -4.29~-0.37; p < 0.001; Figure 6). After excluding QI Y 2024, I 2 = 0 (P = 0.39) (MD = -1.13, 95% CI = -1.50~ -0.76); p < 0.001), indicating that this study is the main source of heterogeneity.

### TNF-a

**Figure 7:**
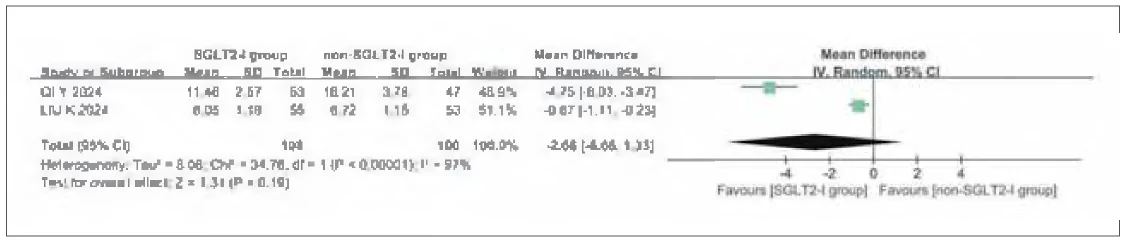
Forest plots regarding TNF-a.

2 studies reported TNF-a, involving 208 patients (108 in the SGLT2-I group and 100 in the non-SGLT2-I group). There was significant heterogeneity between the studies (I^2^ = 97%, P < 0.001), so the random effect model was used for meta-analysis. The results showed that the use of SGLT2-I did not significantly reduce the level of TNF-a (MD = -2.66, 95% CI = -6.66-1.33) compared with the non-use of SGLT2-I. P = 0.19; Figure 7).

### hs-CRP

**Figure 8:**
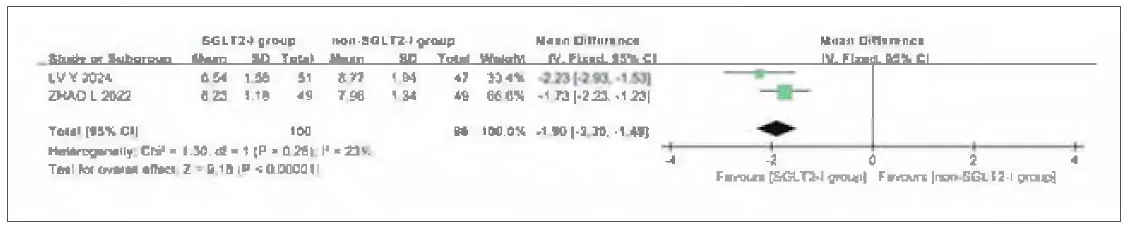
Forest plots regarding hs-CRP.

2 studies reported hs-CRP and a total of 196 patients were included (100 in the SGLT2-I group and 96 in the non-SGLT2-I group). No significant heterogeneity was observed between the studies (I^2^ = 23%, P = 0.25), so the fixed effect model was used for meta-analysis. The results showed that the use of SGLT2-I could significantly reduce the level of hs-CRP (MD = -1.90, 95% CI = -2.30 ~ -1.49; p < 0.001; Figure 8).

### MACE

**Figure 9:**
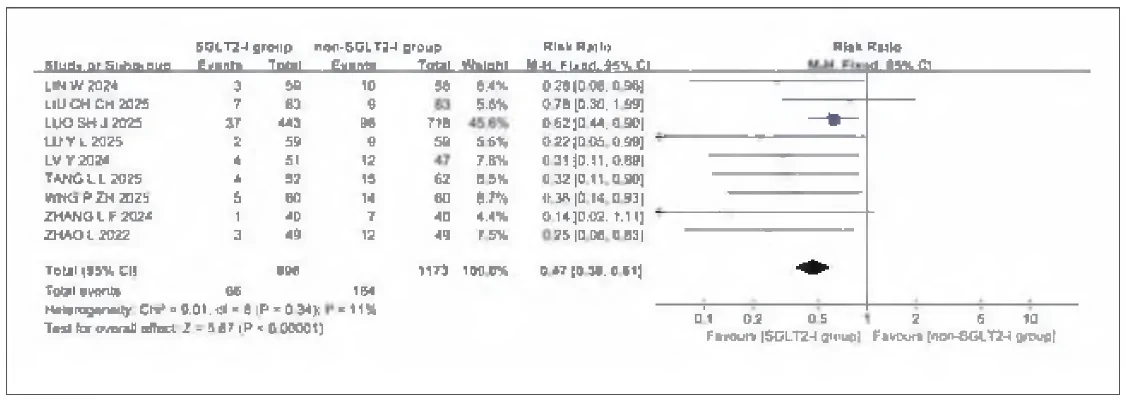
Forest plots regarding MACE.

**Figure 10:**
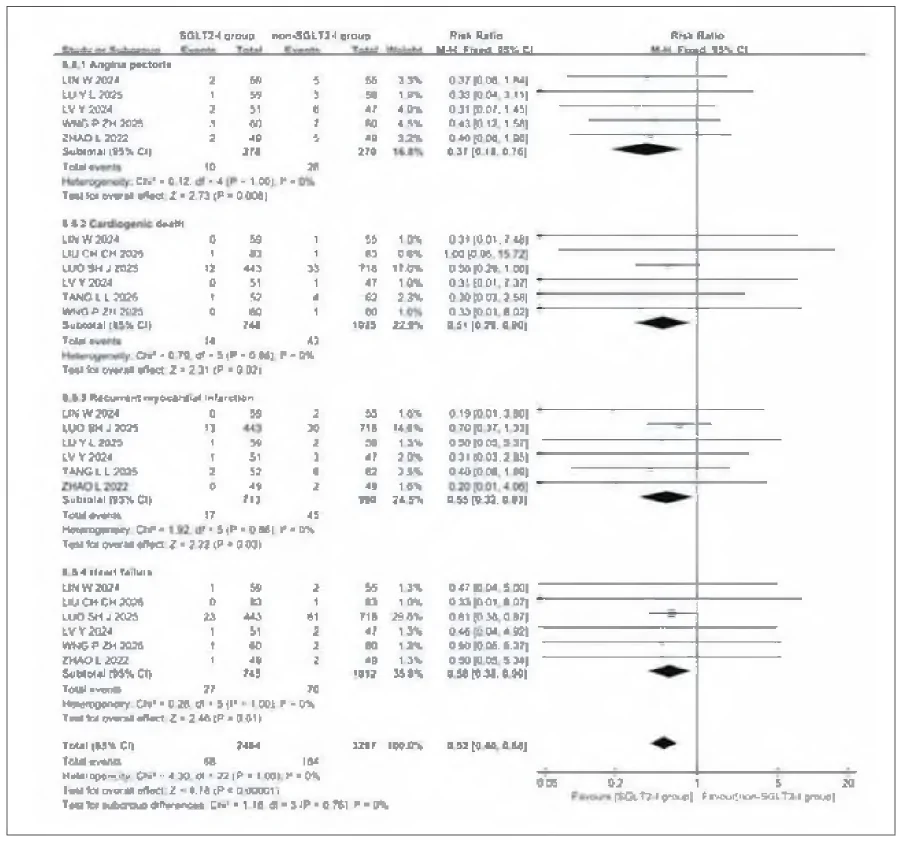
Forest plot of subgroup analysis for MACE components.

Nine studies reported MACE, including 2069 patients (896 in SGLT2-I group and 1173 in non-SGLT2-I group). No significant heterogeneity was observed between studies (I^2^ = 11 %, P = 0.34), so a fixed-effect model was used for meta-analysis. The results showed that the use of SGLT2-I had a lower MACE risk (RR = 0.47,95 % CI = 0.36-0.61) compared with the non-use of SGLT2-I. P < 0.001; Figure 9). A further subgroup analysis of the individual components of MACE demonstrated that SGLT2-I use was associated with a significantly lower risk of angina pectoris (RR=0.37, 95%CI: 0.18-0.76; P=0.006), cardiogenic death (RR=0.51, 95%CI: 0.29-0.90; P=0.02), recurrent myocardial infarction (RR=0.55, 95%CI: 0.32-0.93; P=0.03) and heart failure (RR=0.58, 95%CI: 0.38-0.90; P=0.01) compared with non-SGLT2-I use (Figure 10).

### Publication bias

**Figure 11:**
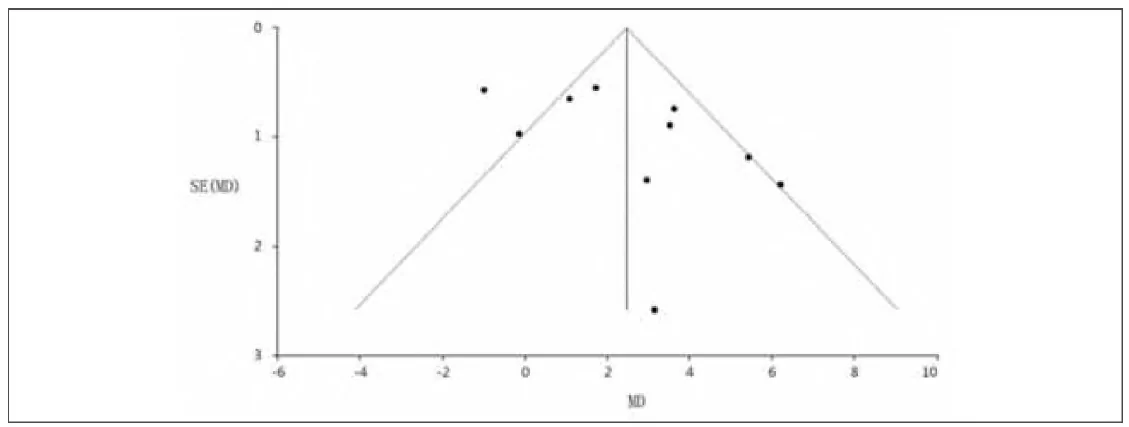
Funnel plot for LVEF.

Publication bias was evaluated for each outcome using Egger's test in conjunction with funnel plots:

LVEF: A total of 10 studies were included. The funnel plot showed a roughly symmetric distribution of effect sizes with no obvious asymmetry (Figure 11); Egger's test yielded a P-value of 0.101, indicating no significant publication bias.

**Figure 12:**
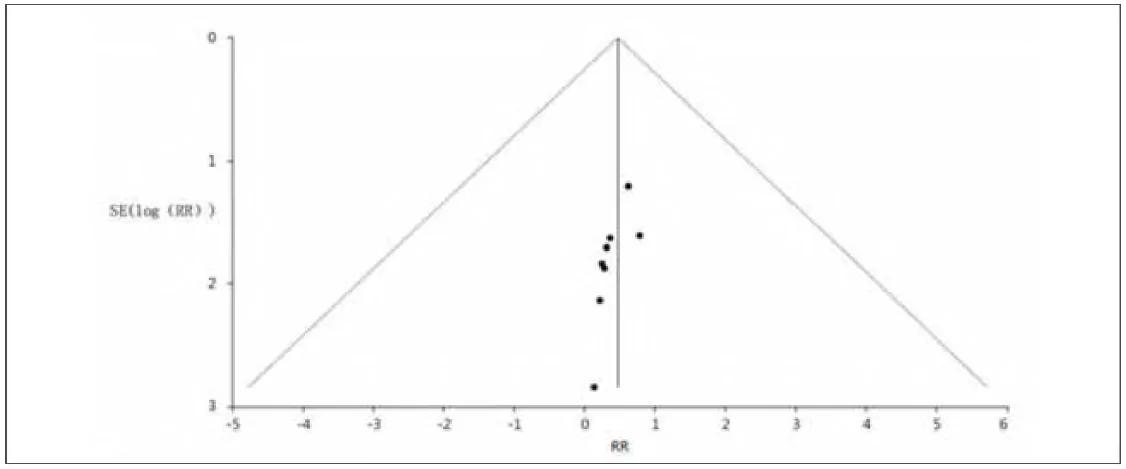
Funnel plot for MACE.

MACE: The funnel plot revealed an asymmetric distribution of study points, suggesting potential publication bias (Figure 12); Egger's test confirmed significant publication bias (P=0.003). Further correction was performed using the Trim-and-Fill method. After imputing 5 hypothetical missing studies, the pooled relative risk (RR) increased from 0.466 to 0.570, which remained below 1 with a 95% confidence interval that did not include 1, suggesting that the conclusion that »SGLT2-I use reduces the risk of MACE« remained robust.

IL-6: Owing to the number of included studies being fewer than 10, only Egger's test was performed (P=0.401), indicating no significant publication bias, and no funnel plot was generated.

### Sensitivity analysis

**Figure 13:**
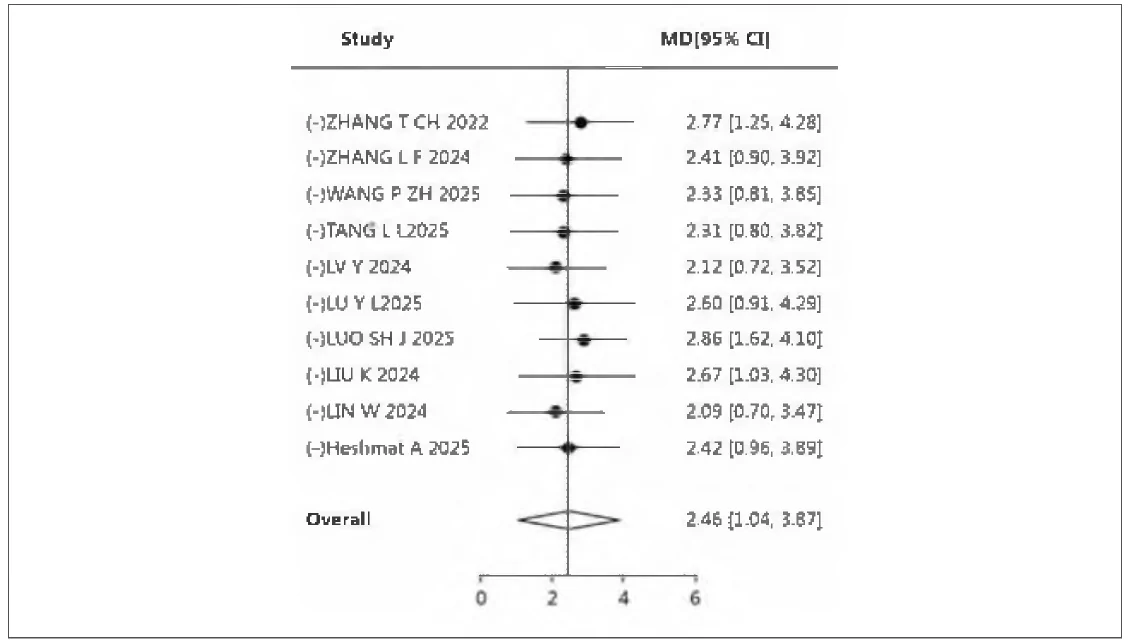
Sensitivity analysis of LVEF.

Sensitivity analysis was performed using the one-by-one elimination method to evaluate the effect of individual studies on the combined effect of LVEF (Figure 13). The results showed that the dominant source of heterogeneity was not identified in this study: after excluding any of the included studies, the heterogeneity test index I^2^ remained above 50%, and the P values corresponding to the combined effect size were all < 0.05. The above results suggest that the Meta-analysis results of LVEF in this study have good robustness.

### GRADE evidence quality rating

GRADE was used to evaluate the quality of evidence for the outcome indicators of the included studies. The results showed that all of them were intermediate quality evidence (Table 2).

**Table 2:** Outcome indicators GRADE evidence quality evaluation results.

Outcome<br>Measure	Quality of Evidence Assessment					Number of Cases		Result	Evidence<br>Grade
	Risk of<br>Bias	Inconsistency	Indirectness	Imprecision	Publication<br>Bias	SGLT2-I<br>group	non-SGLT2-I<br>group		
LVEF	Moderate	Low	None	Low	High	898	1177	MD = 2.46,<br>95%CI = 1.04~3.87	Moderate
IL-6	Moderate	Low	None	Low	Moderate	157	149	MD=-2.33,<br>95%CI=-4.29~-0.37	Moderate
TNF-a	Moderate	Low	None	Low	Moderate	108	100	MD=-2.66,<br>95%CI=-6.66~1.33	Moderate
hs-CRP	Moderate	Low	None	Low	Moderate	100	96	MD=-1.90,<br>95%CI=-2.30~-1.49	Moderate
MACE	Moderate	Low	None	Low	Moderate	896	1173	RR=0.47,<br>95%CI=0.36~0.61	Moderate

## Discussion

AMI is an acute coronary syndrome caused by the rupture of atherosclerotic plaques. It is a chronic inflammatory disease of the arterial wall. Specific cytokines such as IL-6 are involved in this process [22]. Studies have shown that compared with AMI without hyperglycemia, patients with AMI with hyperglycemia have worse cardiac function (reduced LVEF), worse prognosis, and stronger inflammatory response (elevated hs-CRP levels) [23]. This suggests that our inflammatory response may play a more critical role in the pathophysiological process of DM complicated with AMI, and its intensity may be closely related to the degree of cardiac function injury and prognosis. The results of Meta-analysis in this study showed that the use of SGLT2-I could reduce the levels of IL-6 and hs-CRP compared with the non-use of SGLT2-I. Dapagliflozin is a new class of hypoglycemic drugs. As a SGLT2-I, dapagliflozin can not only produce hypoglycemic effect and reduce the effect of hyperglycemia on inflammation, but also reduce the maturation and secretion of inflammatory markers by selectively degrading the inflammasome component NLRP3 [24], which may be the core mechanism of improving the levels of IL-6 and hs-CRP in patients. However, the results of this study showed that the use of SGLT2-I did not show a statistically significant effect in reducing the level of TNF-a compared with the non-use of SGLT2-I. The reason may be related to the high heterogeneity between the studies included in the index, the different types of SGLT2-I, the different treatment duration, and the small amount of included studies.

An increase in LVEF may partially reflect the recovery of myocardial contractile function and indicate improved cardiac pumping capacity, which is of great clinical significance for the evaluation and prognostic assessment of patients with heart failure. The results of this study showed that treatment with SGLT2-I significantly increased LVEF levels compared with no SGLT2-I. The core mechanism is that such agents, represented by dapagliflozin, regulate the reabsorption process in the renal proximal convoluted tubules and promote urinary glucose excretion, thereby effectively reducing blood glucose levels and decreasing cardiac preload, leading to dual benefits of glycemic control and cardiac function improvement. Subgroup analysis indicated that the improvement in LVEF was more significant in studies with a treatment duration of ≥6 months, suggesting that the cardioprotective benefits of SGLT2-I may be time-dependent and require long-term regular administration to achieve full efficacy. However, high heterogeneity remained for this outcome after subgroup analysis, which may be attributed to differences in baseline characteristics of study participants and specific treatment regimens across included studies. Further sensitivity analysis demonstrated that significant heterogeneity persisted after omitting any individual study, but the direction of the pooled effect size was not reversed, confirming the good robustness of the present findings. Nevertheless, since the sources of heterogeneity were not fully identified, the interpretation of these results still requires caution.

MACE caused by AMI are one of the direct causes of death in such patients, and the high incidence of MACE is closely related to myocardial ischemia-reperfusion injury and its mediated chain reactions such as cell injury and cardiac dysfunction [25]. The results of this meta-analysis further indicated that compared with non-SGLT2-I, SGLT2-I administration was associated with a reduced risk of MACEs. This conclusion is consistent with the direction of findings from a meta-analysis conducted by Li Z, et al. [26] which demonstrated that SGLT2-I could effectively decrease all-cause mortality and cardiovascular mortality. The specific mechanisms underlying the beneficial effects of SGLT2-I in the aforementioned patient population have not been fully elucidated. However, studies have confirmed that SGLT2-I exert cardioprotective effects through multiple pathways, including reducing myocardial infarct size, alleviating acute myocardial ischemia/reperfusion injury, and inhibiting inflammation [27]. Therefore, these benefits may stem from two core mechanisms: Glucose-lowering-dependent effects: improving glucose homeostasis to reduce hyperglycemia-mediated myocardial damage and inflammatory responses; Glucose-lowering-independent effects: primarily suppressing the NLRP3 inflammasome to exert anti-inflammatory actions and mitigate myocardial ischemia/reper-fusion injury. These two mechanisms collectively contribute to the improvement of cardiac function and the reduction in MACE risk.

This study has several limitations: First, significant heterogeneity was observed for some outcome indicators. Although subgroup and sensitivity analyses were performed, the sources of some heterogeneity remained unclear. Second, the number of included studies was relatively small, and some were prospective cohort studies. Due to their inherent limitations, caution should be exercised when inferring causal relationships from the conclusions of this study. Third, the dosage and treatment duration of SGLT2 inhibitors varied across the included studies, which may introduce confounding bias in the efficacy evaluation. Fourth, publication bias was detected among studies for some indicators. Although Trim-and-Fill analysis was used for correction, it may still exert a certain impact on the accuracy of the pooled results. Fifth, According to the GRADE system for quality assessment of outcome indicators, all outcomes were rated as moderate-quality evidence. This indicates that the findings of the included studies have certain reference value, but the strength of evidence is limited by factors such as the study design and sample size of the included studies. Therefore, absolutely reliable conclusions cannot be drawn, and more large-sample, high-quality randomized controlled trials are still needed for further verification in the future. Sixth, the included cohort studies did not provide adjusted effect sizes, and only raw data were included for exploratory analysis; therefore, the results should be interpreted with caution. Seventh, only Chinese and English literatures were included in this study, and studies in other languages were not retrieved, which may introduce language bias. The absence of non-Chinese and non-English studies or publication bias of positive results may slightly affect the findings. However, Chinese and English literatures have covered the core research in this field, and the main outcomes were verified to be robust by sensitivity analysis, so the impact of this bias on the study conclusions is limited.

## Conclusions

The results of this meta-analysis showed that SGLT2-I treatment was associated with increased LVEF and decreased levels of IL-6, hs-CRP and MACE risk in DM patients with AMI. In the future, a larger sample size study is still needed to further verify and improve the above conclusions.

## Dodatak

### Authors' contributions

# Jia Xu and Yuanhong Jie contributed equally to this work. Jia Xu, Yuanhong Jie and Xiaobing Liu designed the study and performed the experiments, Tao Dong collected the data, Qi Li analyzed the data, Jia Xu, Yuanhong Jie and Xiaobing Liu prepared the manuscript. All authors read and approved the final manuscript.

### Ethical statement

This study was a meta-analysis based on previously published studies and publicly available data. It did not involve any new experiments on human participants or animals. Therefore, approval from an ethics committee was not required, and informed consent was not applicable. The Helsinki Declaration was not relevant to this study.

### Funding

This study did not receive any funding in any form.

### Conflict of interest statement

All the authors declare that they have no conflict of interest in this work.
